# Effects of photoperiod on life‐history and thermal stress resistance traits across populations of *Drosophila subobscura*

**DOI:** 10.1002/ece3.4945

**Published:** 2019-01-30

**Authors:** Neda N. Moghadam, Zorana Kurbalija Novicic, Cino Pertoldi, Torsten N. Kristensen, Simon Bahrndorff

**Affiliations:** ^1^ Department of Chemistry and Bioscience Aalborg University Aalborg E Denmark; ^2^ Department of Biological and Environmental Science, Centre of Excellence in Biological Interactions University of Jyvaskyla Jyväskylä Finland; ^3^ Animal Ecology, Department of Ecology and Genetics, Evolutionary Biology Center Uppsala University Uppsala Sweden; ^4^ Aalborg Zoo Aalborg Denmark; ^5^ Department of Bioscience Aarhus University Aarhus C Denmark

**Keywords:** adaptation, climate change, *Drosophila*, environmental cues, evolution, photoperiod, plasticity, thermal tolerance

## Abstract

**Introduction:**

Organisms use environmental cues to match their phenotype with the future availability of resources and environmental conditions. Changes in the magnitude and frequency of environmental cues such as photoperiod and temperature along latitudes can be used by organisms to predict seasonal changes. While the role of temperature variation on the induction of plastic and seasonal responses is well established, the importance of photoperiod for predicting seasonal changes is less explored.

**Materials and methods:**

Here we studied changes in life‐history and thermal stress resistance traits in *Drosophila subobscura *in response to variation in photoperiod (6:18, 12:12 and 18:6 light:dark cycles) mimicking seasonal variations in day length. The populations of *D. subobscura* were collected from five locations along a latitudinal gradient (from North Africa and Europe). These populations were exposed to different photoperiods for two generations, whereafter egg‐to‐adult viability, productivity, dry body weight, thermal tolerance, and starvation resistance were assessed.

**Results:**

We found strong effects of photoperiod, origin of populations, and their interactions on life‐history and stress resistance traits. Thermal resistance varied between the populations and the effect of photoperiod depended on the trait and the method applied for the assessment of thermal resistance.

**Perspectives:**

Our results show a strong effect of the origin of population and photoperiod on a range of fitness‐related traits and provide evidence for local adaptation to environmental cues (photoperiod by population interaction). The findings emphasize an important and often neglected role of photoperiod in studies on thermal resistance and suggest that cues induced by photoperiod may provide some buffer enabling populations to cope with a more variable and unpredictable future climate.

## 1 INTRODUCTION

Ectotherms must cope with daily and seasonal changes in environmental conditions (Bahrndorff, Loeschcke, Pertoldi, Beier, & Holmstrup, 2009; Cossins & Bowler, 1987; Dahlhoff & Rank, 2007), among which temperature extremes and lack of adequate nutrition can affect population viability and individual fitness (Andersen, Kristensen, Loeschcke, Toft, & Mayntz, 2010; Braby & Jones, 1995; Dahlhoff & Rank, 2007; Fischer & Fiedler, 2001). Variation in environmental conditions in nature can be dramatic, and it is predicted that the magnitude and frequency of extreme local weather events will increase in the coming decades due to global climate changes (IPCC, 2013). Species can cope with such stressful environments through plastic and/or evolutionary responses leading to, for example, behavioral, morphological, and/or physiological adjustments (Hoffmann & Parsons, 1991). These processes occur at different timescales, and their efficacy rely on the predictive value of environmental cues that trigger the response (Kristensen, Ketola, & Kronholm, 2018).

Temperature and photoperiod are environmental factors providing signals that regulate the induction of stress responses (Teets & Denlinger, 2013; Tyukmaeva, Salminen, Kankare, Knott, & Hoikkala, 2011; Williams & Sokolowski, 2009). For example, hardening or acclimation responses induced by thermal variation can modulate resistance to temperature extremes as well as impact upon starvation and desiccation resistance (Alemu, Alemneh, Pertoldi, Ambelu, & Bahrndorff, 2017; Parkash, Aggarwal, Singh, Lambhod, & Ranga, 2013; Schou, Loeschcke, & Kristensen, 2015). The evolutionary importance of physiological processes as a consequence of variation in ambient temperature is strongly supported by data from clinical studies, where latitudinal gradients in thermal conditions have resulted in intraspecific clinal variation in stress resistance (Castaneda, Rezende, & Santos, 2015; Hoffmann, Anderson, & Hallas, 2002; Kingsolver & Buckley, 2017; Pratt & Mooney, 2013; Yampolsky, Schaer, & Ebert, 2014). These findings demonstrate the importance of past evolutionary processes for contemporary ecological dynamics (see Hoffmann et al., 2002 for a review).

Apart from thermal variation, photoperiod can play an important role in mediating seasonal events and stress resistance. The timing of migration in many species of birds, range expansion of species across latitudes, or flowering time in plants all happen partly in response to seasonal changes in day length (Itoh & Izawa, 2013; Pulido, Coppack, & Berthold, 2001; Saikkonen et al., 2012). The modulatory effect of photoperiod on thermal resistance has been reported in various species of *Drosophila* (Hoffmann, Shirriffs, & Scott, 2005; Lanciani, Giesel, Anderson, & Emerson, 1990; Vesala & Hoikkala, 2011). For example, in *Drosophila melanogaster*, a shorter photoperiod reduced the cold resistance of adult flies (Bauerfeind, Kellermann, Moghadam, Loeschcke, & Fischer, 2014; Hoffmann et al., 2005), which was in contrast to previous studies on other *Drosophila* species (Hori & Kimura, 1998; Lanciani, Lipp, & Giesel, 1992; Vesala, Salminen, Kankare, & Hoikkala, 2012). The importance of photoperiod and stress resistance has also been investigated in a lowland population of *Drosophila buzzatii*, in which different light regimes changed the heat knock‐down resistance so that flies increased resistance during the hours of the day with light where they were most active and thermal conditions were most favorable (Sørensen & Loeschcke, 2002). This was interpreted as an adaptation to surviving the warm conditions experienced by lowland populations. Interestingly, the importance of photoperiod was not observed in a nearby highland population generally experiencing lower temperatures. The species‐ and population‐specific effects of photoperiod suggest that responses have a genetic basis and/or can be affected by assay conditions (Williams & Sokolowski, 2009). Apart from the effects of photoperiod on thermal resistance, Fischer et al. (2012) showed that photoperiod is important for a range of life‐history traits including growth rate, development time, and body size in *Protophormia terraenovae* and for many of the traits investigated results showed interactions between temperature and photoperiod.

In contrast to temperature, photoperiod can be defined as a highly reliable environmental cue due to the constant variations in photoperiod across days and seasons for a given latitude and/or altitude (Bradshaw & Holzapfel, 2007; Jackson, 2009; Salis, van den Hoorn, Beersma, Hut, & Visser, 2018). The day length that triggers the incidence of seasonal activities (critical photoperiod) is linked to the length of the growing season and the timing of the onset of a specific season. Therefore, in the northern hemisphere at northern latitudes, where winter sets in earlier and the growing season is shorter than in the south, organisms use a longer critical photoperiod as a cue to switch between seasonal phenotypes (Bradshaw, 1976; Bradshaw & Holzapfel, 2007; Danilevskiĭ, 1965). Further, a reduction in the critical photoperiod of populations inhabiting the northern latitudes, due to the later onset of winter and a longer growing season caused by climate change, has been observed (Bradshaw & Holzapfel, 2006). For example, the pitcher‐plant mosquito *Wyeomyia smithii *has over a period of 24 years (from 1972 to 1996) shifted toward shorter photoperiods to initiate the larval dormancy (Bradshaw & Holzapfel, 2001). These results show the adaptive potential of photoperiodism and the importance of photoperiod in relation to coping with climate change through evolutionary responses.

Despite the importance of day length for multiple life‐history traits and for the ability to cope with stressful thermal conditions, most experimental studies investigating the ability to perform in environments with variable temperatures have neglected the role of photoperiod and its importance for stress resistance and for shaping the distribution of species (Ketola & Saarinen, 2015; Kristensen, Kjeldal, Schou, & Lund, 2016; Manenti, Sørensen, Moghadam, & Loeschcke, 2014, 2016). In the present study, we investigate the importance of seasonal variation in day length on stress resistance and life‐history traits of five populations of *Drosophila subobscura *distributed across North Africa and Europe spanning the latitudes 31°N to 59°N (Table 1). The aim of the study was to evaluate to what extent populations from different latitudes differ in photoperiod‐associated responses. We hypothesize that short day length increases cold resistance and long day length increases heat and starvation resistance (Hoffmann et al., 2005; Lanciani et al., 1990, 1992). We expect that a long day length reduces energy invested in reproduction and increases energy used for building up body reserves and for inducing stress responses. We further hypothesize that photoperiod‐dependent responses are more pronounced for high‐latitude populations evolved to cope with highly variable and unpredictable thermal environments (Bahrndorff et al., 2009; Sniegula, Nilsson‐Örtman, & Johansson, 2012).

**Table 1:** Position, number of collected inseminated females used to establish each population, year of collection and annual minimum and maximum daylight period for the populations

ID	Population	Position	Location	Inseminated females	Year (month)	Min. daylight (min)	Max. daylight (min)
MO	Morocco	31°11′N, 8°15′W	Amizmiz	15	2016 (Jul)	606	852
SP	Spain	41°43′N, 2°12′E	Font Groga	20	2015 (Oct)	552	909
SE	Serbia	43°33′N, 20°45′E	Mountain Goc	50	2015 (Jun)	534	928
DE	Denmark	55°56′N, 10°12′E	Odder	14	2016 (Jan)	414	1,057
SW	Sweden	59°49′N, 17°54′E	Fjällnora	35	2016 (Jul)	358	1,125

## 2 MATERIALS AND METHODS

### 2.1 Fly populations

**Figure 1:**
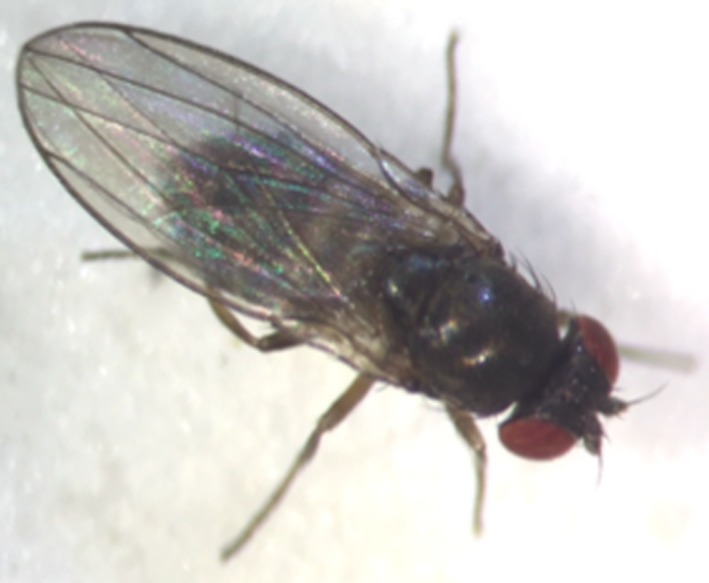
Female fly of *Drosophila subobscura*

The experiments were performed on *D. subobscura *(Figure 1), a Palearctic species with a wide distribution including North Africa, southern Europe, and Scandinavia (Krimbas, 1993). The European populations of *D. subobscura* have been shown to be genetically variable, and the species is an excellent model in evolutionary studies (Pascual et al., 2007). This species does not have a photoperiodically controlled reproductive diapause for overwintering (Goto, Yoshida, Beppu, & Kimura, 1999), which is an advantage when studying the impact of photoperiod on fitness components using a common garden set up.

**Figure 2:**
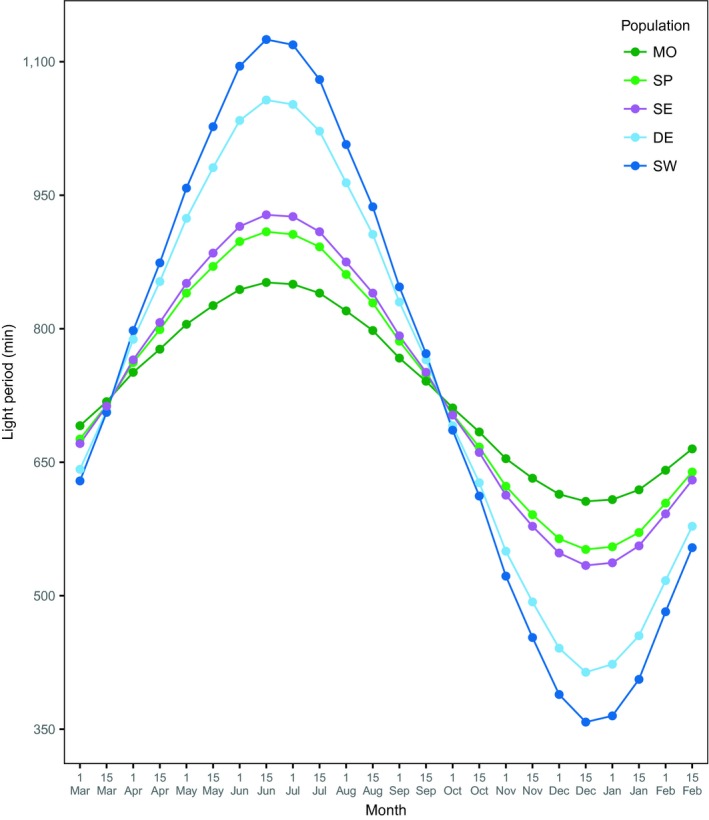
Seasonal variations in day length (min) for each of the sampling locations (MO: Morocco, SP: Spain, SE: Serbia, DE: Denmark and SW: Sweden). The graphs are based on 24 time points per location (six points per season) and represent latitudinal variations in the amplitude of photoperiod

The study populations used in the present study were collected from five different locations. These locations differ in photoperiod throughout the year (Figure 2). The sampling locations, year of collection, number of inseminated female flies used to establish the populations, and annual minimum and maximum of daylight are presented in Table 1. After collection, each population was kept as one interbreeding mass population in 300‐ml plastic bottles (4 bottles per population, ca. 500 individuals per bottle) containing 70‐ml standard *Drosophila* agar–sugar–yeast medium seeded with live yeast and maintained under laboratory condition for approximately one year before starting the experiment. For the purpose of the experiment, the populations were kept at 23°C under three different light regimes (6:18, 12:12 and 18:6 light:dark cycles with lights on at 9 a.m.) for two generations. Throughout the experiment, the flies were maintained at 23°C (unless otherwise stated), which is favorable for numerous fitness components in this species (Santos, 2007). In the second generation, the eclosed flies from each population and light regime were collected within 48 hr of eclosion and placed in 300‐ml plastic bottles (ca. 100 individuals per bottle) containing 70‐ml standard *Drosophila* medium enriched with live yeast to increase females’ fecundity. Each day adult flies (aged 5–6 and 8–9 days for the first and last block, respectively) were given 16 hr to lay eggs on small spoons filled with 1‐ml *Drosophila* standard medium seeded with live yeast. The collection of eggs was conducted at a controlled density (see below) in four consecutive blocks (one block per day) for logistic reasons.

### 2.2 Traits assessed

#### 2.2.1 Egg‐to‐adult viability

Eggs were collected at a controlled density (25 eggs per vial) and placed into 35‐ml plastic vials (12 replicates per population per light regime for each block) containing 7‐ml standard *Drosophila* medium. The eggs developed at the respective light regime experienced by their parents under controlled conditions (23°C, 60% humidity). Following the first eclosion, number of eclosing flies was scored every 24 hr until all the flies had emerged. The flies stuck in the medium were scored as alive and removed using a brush. The flies collected within the first 24 hr were transferred to 35‐ml plastic vials (25 ± 5 flies per vial) with 3‐ml standard *Drosophila* medium. Unless otherwise stated, at 3 days of age flies were separated by sex under light CO_2_ anesthesia and males were placed at a controlled density (25 ± 5 males per vial) in 35‐ml plastic vials with 3‐ml standard *Drosophila* medium and placed in the respective light regime for further assessments.

#### 2.2.2 Productivity

To assess the productivity of flies exposed to different light regimes, one male and one female fly at 3 days of age were placed in 35‐ml plastic vials (20 vials per population per light regime were divided equally into four blocks) with 10‐ml standard *Drosophila* medium without live yeast. Every 72 hr, the pairs were transferred to new vials with fresh food using an aspirator to prevent losing flies. This process continued for 9 days (from 3 to 12 days of age). The vials were kept at their respective light regime under controlled condition (23°C, 60% humidity). The number of offspring produced per pair was recorded after eclosion until all the flies had emerged.

#### 2.2.3 Dry body weight

Male flies were dried at 60°C for 24 hr and thereafter placed in a desiccator. The dry body weight (dbw) of flies was measured in batches of four males (9 batches per population per light regime) to a precision of 0.01 mg using a Sartorius® electrobalance (Quintix 35–1S, Germany).

#### 2.2.4 Thermal resistance assay

To assess critical thermal maximum (CTmax) and critical thermal minimum (CTmin) of flies from the five populations and three experimental treatments, 5‐day‐old males (20 males per population per light regime were divided with five flies in each of four blocks) were placed individually into 5‐ml screw cap glass vials without the use of anesthetics. The vials were placed randomly in a metal rack and then submerged in a water bath set at 23°C. Thereafter, the water temperature was increased (to assess CTmax) or decreased (to assess CTmin) with a rate of 0.1°C/min and the temperature at which a fly was totally immobilized was scored until all flies were in coma.

#### 2.2.5 Locomotor activity under heat ramping

The locomotor activity of 5‐day‐old males was recorded using an assay where temperature was gradually increased (ramped up). Flies were placed in *Drosophila* Activity Monitors (DAM; Trikinetics, Waltham, MA, USA) in which infrared detectors registered an activity count each time a fly crossed a beam (Pfeiffenberger, Lear, Keegan, & Allada, 2010). Each trial started at 10 a.m. when flies (60 males per population per light regime divided with 15 in each of four blocks) were individually placed into a narrow glass tube (5 mm ×65 mm) with parafilm at one end and a wet cotton stopper at the other end to prevent desiccation, but allowing some air ventilation. The glass tubes were placed in the DAMs randomly, where each monitor contained two samples of each light regime and population. Around 11 a.m., the DAMs were placed in the incubator set at 23°C for 1 hr followed by an increase in temperature with a rate of 0.1°C/min until the temperature reached 40°C. The humidity was kept at 60% in the incubator, and the assay was performed at constant light. The activity was recorded every 30 s, and sum of the activity counts was calculated as the total locomotor activity (TLMA) for an individual. In addition, we recorded the temperature at which no further activity (CTmax_(LMA)_) was observed for each individual.

#### 2.2.6 Starvation resistance

Male flies at 5 days of age from each population and light regime were placed individually in 35‐ml plastic vials (20 males per population per light regime were divided with five flies in each of four blocks) containing 2 ml of agar–water medium (20 g/L) to provide moisture and avoid desiccation during the experiment. The vials were kept at the respective light regime experienced by parents under controlled conditions (23°C, 60% humidity). The number of dead flies was scored every 12 hr until all flies had died.

### 2.3 Statistical analyses

Prior to analysis all data were tested for normality and homogeneity of variance, and the block effect was adjusted by a standardization using the grand mean. To obtain normally distributed data and homogeneity of variance, the TLMA and CTmax_(LMA)_ were log and square root transformed, respectively. The effects of population, photoperiod, and their interaction on various traits (viability, total productivity, DBW, and thermal and starvation resistance) were tested using a two‐way ANOVA with population and photoperiod as fixed factors. The unit of replication was group of flies (eggs or adults) for viability (a group consisted of 25 eggs), productivity (a group consisted of one male and one female), and DBW (a group consisted of four males) and individual flies for the remaining traits. Populations were treated as a fixed effect as our main focus was on the variance between populations. The productivity of flies was analyzed by repeated measures ANOVA to correct for repeated measurements from the same individual. In all significant cases, multiple pairwise comparisons were performed using Tukey's method. All statistical analyses were performed in R, version 3.5 and R studio, version 1.1.44 (R Core Team, 2017).

## 3 RESULTS

### 3.1 Life‐history traits

#### 3.1.1 Egg‐to‐adult viability

**Figure 3:**
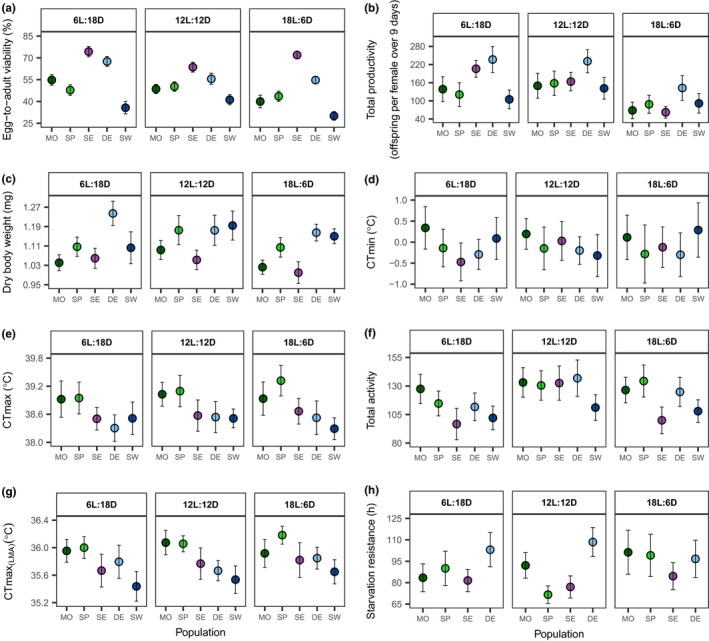
Variation in (a) egg‐to‐adult viability (percentage of eggs developing into adult flies); (b) total productivity; (c) dry body weight; (d); CTmin; (e) CTmax; (f) total activity (sum of locomotor activity under a ramping temperature from 23°C to 40°C with 0.1°C increase per minute); (g) CTmax_(LMA)_ (the highest temperature with no scored activity afterward) and (h) starvation resistance of five populations (MO: Morocco, SP: Spain, SE: Serbia, DE: Denmark and SW: Sweden) in response to three different light regimes (6L:18D, 12L:12D and 18L:6D). Symbols indicate mean (95% CI)

Egg‐to‐adult viability of flies varied between populations (*F*_4, 720_ = 170.53, *p* < 0.0001; Table 2). In general, the SE (69.97%) and SW (35.64%) populations showed the highest and lowest viability, respectively (Figure 3a). The viability of the SP and MO populations was similar (47.79% and 47.22%, respectively) and significantly (*p* < 0.0001) lower than the viability of the DE population (59.28%). On average, egg‐to‐adult viability decreased significantly when we increased the day length (*F*_2, 720_ = 26.49, *p* < 0.0001). There was a significant interaction between photoperiod and population (photoperiod × population: *F*_8, 720_ = 8.41 and *p* < 0.0001; Table 2) indicating that photoperiod had distinct impacts on viability in populations from different latitudes. The egg‐to‐adult viability of the MO, SP, and SW populations exposed to the 12L:12D photoperiod was considerably higher than the corresponding populations under the 18L:6D light regime (MO: *p* = 0.004, SP: *p* = 0.02, SW: *p* < 0.0001). Moreover, the viability of the MO population under the 6L:18D photoperiod was approximately 15% higher than the 18L:6D treatment group (*p* < 0.0001). In the DE population, no difference was observed in the viability of the 12L:12D and the 18L:6D treatment groups (*p* > 0.05) and the highest viability was observed in the 6L:18D group (6L:18D vs. 12L:12D, *p* < 0.0001; 6L:18D vs. 18L:6D, *p* < 0.0001). The viability of the SE population under the 6L:18D or 18L:6D treatment was, respectively, similar or higher than the 12L:12D group (6L:18D vs. 12L:12D, *p* < 0.0001; 18L:6D vs. 12L:12D, *p* < 0.001, 6L:18D vs. 18L:6D, *p* > 0.05).

**Table 2:** Results of the overall ANOVA analysis to examine the effect of population, light regime, and their interaction on egg‐to‐adult viability, productivity, DBW (dry body weight), CTmin and CTmax (thermal resistance), TLMA (total locomotor activity), CTmax_(LMA)_ (the highest temperature with no observed activity at higher temperatures), and starvation resistance. Significant *p* values (*p* < 0.05) are represented in bold

Trait	Source	*df*	Sum of square	*F*	*p*‐value
Viability	Pop	4	98,540	170.53	**<0.0001**
	Light	2	7,653	26.49	**<0.0001**
	Pop × Light	8	9,715	8.41	**<0.0001**
Productivity	Pop	4	332,222	14.80	**<0.0001**
	Light	2	372,122	33.16	**<0.0001**
	Pop × Light	8	111,053	2.47	**0.01**
DBW	Pop	4	0.63	30.28	**<0.0001**
	Light	2	0.07	7.27	**0.0009**
	Pop × Light	8	0.11	2.77	**0.006**
CTmin	Pop	4	6.85	2.36	0.05
	Light	2	0.05	0.03	0.97
	Pop × Light	8	5.09	0.88	0.54
CTmax	Pop	4	22.70	12.80	**<0.0001**
	Light	2	0.79	0.89	0.41
	Pop × Light	8	2.14	0.60	0.77
TLMA	Pop	4	6.98	9.96	**<0.0001**
	Light	2	3.46	9.90	**<0.0001**
	Pop × Light	8	2.41	1.72	0.09
CTmax_(LMA)_	Pop	4	0.22	14.66	**<0.0001**
	Light	2	0.02	2.84	0.06
	Pop × Light	8	0.02	0.86	0.55
Starvation	Pop	3	15,107	9.18	**<0.0001**
	Light	2	2,800	2.55	0.08
	Pop × Light	6	10,180	3.09	**0.006**

#### 3.1.2 Productivity

Productivity differed between populations (*F*_4, 285_ = 14.80, *p* < 0.0001; Table 2). The highest productivity was observed in the DE population (Figure 3b). In general, exposure to long hours of daylight reduced the total productivity of populations (*F*_2, 285_ = 33.16, *p* < 0.0001). This reduction was more pronounced under the 18L:6D photocycle. The influence of photoperiod on flies’ productivity differed between populations (photoperiod × population: *F*_8, 285_ = 2.47, *p* < 0.01). The DE and SE populations showed a constant reduction in the total productivity with increasing day length, while the productivity of the MO, SP, and SW populations increased at the 12L:12D and reduced at the 18L:6D photoperiod. Repeated‐measure ANOVA with population, photoperiod, and age as crossed fixed factors revealed a significant effect of age on the productivity of populations developed under the three different light regimes (*F*_3, 283_ = 58.18, *p* < 0.0001).

#### 3.1.3 Dry body weight

Population (*F*_4, 178_ = 30.28, *p* < 0.0001), photoperiod (*F*_2, 178_ = 7.27, *p* = 0.0009), and their interaction (photoperiod × population: *F*_8, 178_ = 2.77, *p* = 0.006) affected the Dry body weight (DBW) of flies (Table 2). Across different photoperiods, the MO and SE populations showed the lowest and the DE population the highest DBW (Figure 3c). In general, the DBW of flies kept at 6L:18D and 18L:6D was similar except for the DE population in which the flies exposed to the 6L:18D treatment were approximately 6.6% (*p* = 0.04) heavier than flies from the 18L:6D group. The development of the MO and SW populations under a 12L:12D photoperiod significantly increased the DBW of flies compared to the 18L:6D (*p* < 0.01) and 6L:18D (*p* < 0.05) light regimes. The photoperiod did not affect the DBW of the SE population (*F*_2, 35_ = 2.85, *p* = 0.07).

### 3.2 Stress resistance

#### 3.2.1 Thermal resistance

Neither population nor photoperiod affected the cold resistance (CT_min_) of flies (Figure 3d). There was a significant effect of population on CT_max_ (*F*_4, 293_ = 12.80, *p* < 0.0001; Table 2); the SP and MO populations showed a higher heat resistance than the other three populations (Figure 3e). There was no effect of photoperiod on CT_max_ (*F*_2, 293_ = 0.89, *p* > 0.05; Table 2).

#### 3.2.2 Locomotor activity during heat ramping

The total locomotor activity (TLMA) of flies during heat ramping differed between populations (*F*_4, 869_ = 9.96, *p* < 0.0001; Table 2) with flies from the SE and SW populations being least active. The difference in the TLMA of populations was more pronounced during the 6L:18D and 18L:6D photoperiods (Figure 3f). There was also a significant effect of photoperiod on TLMA (*F*_2, 869_ = 9.90, *p* < 0.0001), where the lowest activity was observed in the 6L:18D treatment groups. The pairwise comparisons showed no difference in activity between photoperiods for the MO (*F*_2, 172_ = 0.21, *p* > 0.05), SP (*F*_2, 176_ = 2.54, *p* > 0.05), DE (*F*_2, 170_ = 2.50, *p* > 0.05), and SW (*F*_2, 176_ = 0.66, *p* > 0.05) populations. However, the SE population under the 12L:12D photoperiod showed higher activity compared to 6L:18D (*p* < 0.001) and 18L:6D (*p* < 0.01) photoperiod. The CTmax_(LMA)_ differed between populations (*F*_4, 882_ = 14.66, *p* < 0.0001; Table 2) with the MO and SP populations being the most heat resistant and SW the least heat resistant population (Figure 3g). This pattern was present for both the 6L:18D and 12L:12D photoperiod treatment groups, but with the MO population being less heat resistant for the 18L:6D treatment group.

#### 3.2.3 Starvation resistance

The population from Sweden (SW) was removed from the data set due to the small sample size at 18L:6D photoperiod. The overall analysis displayed significant effects of population (*F*_3, 227_ = 9.18, *p* < 0.0001) and the interaction with photoperiod (*F*_6, 227_ = 3.09, *p* < 0.01) on the starvation resistance of flies (Table 2). Across all treatments, the starvation resistance of the DE population was significantly higher than in other populations, except at the 18L:6D photocycle where no difference was observed between populations (*F*_3, 77_ = 1.36, *p* > 0.05; Figure 3h). The effect of photoperiod on the starvation resistance of populations was not significant (*F*_2, 227_ = 2.55, *p* > 0.05), except for the SP population in which exposure to 18L:6D photoperiod increased the starvation resistance of individuals compared to the 12L:12D treatment group (*p* < 0.01, ca. 28 hr).

## 4 DISCUSSION

Organisms respond to changes in photoperiod via plastic and evolutionary responses. These responses have been proposed to be essential for survival and reproductive success of organisms in seasonal environments (Bradshaw & Holzapfel, 2007; Kimura & Beppu, 1993; Tyukmaeva et al., 2011). The contribution of day length in mediating the optimal timing of seasonal events varies as a function of latitude. Reliance on photoperiod elevates with increasing distance from equator and is typically more pronounced in populations inhabiting the northern latitudes (Sniegula et al., 2012), which also suggests that genotype‐by‐photoperiod interactions are important for the expression of seasonal phenotypes. In most studies on thermal adaptation, the importance of photoperiod is rarely considered and the main focus in such studies is on the role of thermal changes in mediating thermal resistance, life‐history traits, and in shaping the distribution of species (Ketola & Saarinen, 2015; MacLean, Kristensen, Overgaard, Sørensen, & Bahrndorff, 2017).

In the present study, we observed photoperiod‐associated changes in life‐history and thermal stress resistance traits across various populations of *D. subobscura*. Overall, the results show that the photoperiodic regulation of the flies’ phenotypic performance was trait and population specific.

Populations used in our study have been kept in the laboratory for several generations before performing the experiments presented in this paper. We acknowledge challenges related to this; that is, potential laboratory adaptation and genetic drift. However, the populations were founded based on a large number of individuals and kept at a large population size (more than 2,000 individuals per population). Therefore, it is unlikely that the patterns established are caused by chance events like genetic drift or bottleneck. Further, we argue that even though the populations had been kept in the laboratory for up to one year prior to the experiment, findings show that laboratory maintenance does not affect fundamental species characteristics and validates comparative studies based on laboratory maintained populations (Maclean, Kristensen, Sørensen, & Overgaard, 2018).

### 4.1 Effect of photoperiod and population on stress resistance

The results of the present study show that stress resistances (cold, heat, and starvation resistance) differed between populations of *D. subobscura*. Generally, the most southern populations were most heat resistant and the northern populations most cold resistant. This is in accordance with other studies looking at thermal adaptation across populations and indicates the presence of a geographic thermal tolerance cline for this species (Castaneda et al., 2015; David, Gibert, Moreteau, Gilchrist, & Huey, 2003). However, some populations deviated from this pattern potentially due to the influence of the applied photoperiods. For example, the SW population exposed to a long photoperiod is less cold resistant compared to the other populations (Figure 3d), which may signify the higher reliance of this northernmost population on day length for adjusting their seasonal phenotypes. This suggests that photoperiod‐related plasticity appears particularly important for populations living in highly fluctuating and unpredictable thermal environments.

Data on starvation resistance also showed evidence for local adaptation with a population‐specific pattern as indicated by the significant interaction between population and photoperiod. This is in agreement with Gilchrist et al. (2008), where male *D. subobscura* collected across Europe showed large differences in starvation resistance between geographical areas. The linear cline for starvation resistance observed at a short photoperiod follows our expectation of higher starvation resistance with increasing latitude since northern populations may face food deprivation during cold seasons. However, this result is in contrast with previous findings where starvation resistance decreased with increasing latitude (Arthur, Weeks, & Sgrò, 2008; Hoffmann, Hallas, Sinclair, & Mitrovski, 2001; Sisodia & Singh, 2010). This inconsistency may arise from the influence of photoperiod on the association between starvation resistance and latitude, which suggests that selection on this trait is inconsistent across seasons and stronger in seasons with short photoperiods. It also demonstrates the considerable plasticity of starvation resistance in response to environmental cues (Rion & Kawecki, 2007).

We scored behavioral performance (locomotor activity) under heat ramping to estimate the critical upper thermal limits of flies. The assessment of thermal resistance through behavioral performance allowed us to track the locomotor activity of flies throughout heat ramping and use a less invasive method to estimate the temperature at which activity is no longer recorded. Both total activity and CTmax_(LMA)_ were affected by population, and there was a significant effect of photoperiod on total locomotor activity recorded throughout the heat ramping assay (Figure 3f and G). In general, the high activity of populations exposed to a long photoperiod prior to entering the heat ramping assay supports the general notion that activity of flies is higher during the growing season when the temperature is high (Wolda, 1988). Moreover, the photoperiod‐related increase in the total activity highlights that this trait is highly plastic which may be relevant for explaining the global distribution of this species (Prevosti et al., 1988). Although not significant, individuals reared at 18:6 L:D showed higher CTmax_(LMA)_ values, which suggest an improvement in thermal tolerance in response to long day length, which is in agreement with the findings of Fischer et al. (2012).

### 4.2 Effect of photoperiod and population on life‐history traits

Interestingly, photoperiod affected all life‐history traits investigated in the present study. Interpreting the differences in response to photoperiod can be challenging as treatments represent a mix of conditions, and their relevance depend on the geographic origin of the population in question and the sensitivity and flexibility of the trait. For example, the egg‐to‐adult viability of populations showed an overall increase with latitude, although the SW population deviated from this pattern (Figure 3a). A positive correlation between viability and latitude has been observed in *D. buzzatii *(Folguera, Ceballos, Spezzi, Fanara, & Hasson, 2008), but negative or no correlation has also been found (Loeschcke, Bundgaard, & Barker, 2000; Overgaard, Kristensen, Mitchell, Cockerell, & Hoffmann, 2010). The low viability of the SW population specially at the 18L:6D photoperiod may arise from photoperiod‐induced disruption of the circadian system due to the higher plasticity in this northern population (Liu & Zhao, 2014). Clinal variation in phenotypic plasticity is observed in many organisms, and this might be explained by higher fluctuations in environmental conditions at northern latitudes (Li, Du, Guan, Yu, & van Kleunen, 2016; Mathur & Schmidt, 2017; Molina‐Montenegro & Naya, 2012). A reduction of viability in response to day length has been observed in *D. melanogaster*, where a long photoperiod reduced survival (Vaiserman et al., 2008). The biological reason behind the photoperiod‐related reduction in viability is currently unclear.

The total productivity reduced with increasing day length in a population‐specific manner; that was more pronounced in the SE population (Figure 3b). The negative influence of long photoperiod on productivity has been observed also in *Sesamia nonagrioides,* where long day length suppressed the oviposition of this insect (Fantinou, Perdikis, & Zota, 2004). In another study by Hodek and Iperti (1983), exposure to short day length increased the females’ fecundity of *Semiadalia undecimnotata*. Therefore, the tendency of females to lay more eggs at shorter day length can partly explain the differences in productivity between populations (Fluegel, 1981; Oshima, Inoue, & Ishiwa, 1972; Shakunthala, 2014). In addition, the low egg‐to‐adult viability of populations under long day length can affect the reproductive potential of populations.

The DBW of populations displayed a positive trend with increasing latitude (Figure 3c), which is in agreement with a previous study on European population of *D. subobscura* (Gilchrist, Huey, & Serra, 2001). According to the temperature–size rule, ectotherms usually get smaller with an increase in the developmental temperature (Atkinson, 1994; Walters & Hassall, 2006). Therefore, as expected flies from MO and northern Europe had the lowest and the highest DBW respectively, although this was not the case for the SW population at the 6L:18D photoperiod. Results obtained on other diptera species (*Musca domestica*) have shown similar results, where body size to some degree is independent of latitude (Bryant, 1977; Kjærsgaard, Blanckenhorn, Pertoldi, Loeschcke, & Bahrndorff, 2015). Among the light regimes, the highest DBW of populations was observed at the 12L:12D photoperiod except for the DE population. This finding is in agreement with the study on the northern damselfly (*Coenagrion johanssoni*) in which the central European populations showed the highest growth rate during spring (Sniegula et al., 2012).

## 5 CONCLUSION

Our results revealed a significant effect of population and photoperiod on a range of fitness‐related traits in *D. subobscura*. However, photoperiod‐related responses varied between traits which may be explained by different sensitivity and plastic abilities. The observation of better performance of the *D. subobscura* at short and medium photoperiods is in accordance with the high abundancy of this species in early spring in nature and its high cold tolerance (Budnik & Brncic, 1983; Sørensen, Kristensen, Loeschcke, & Schou, 2015). The presence of strong local adaptation to day length (photoperiod by population interaction) highlights the importance of photoperiod as a selective agent in the study of population differences observed in fitness traits. However, further experiments are needed to fully understand the evolutionary significance of photoperiod in mediating seasonal events across populations. For example, day lengths mimicking natural conditions for each population should be incorporated into future studies to fully assess the geographic cline in photoperiodism.

In conclusion, the results of the present study emphasize the role of photoperiod in evaluating the consequences of fast environmental changes and suggest that cues induced by photoperiod may provide some buffer enabling populations to cope with a more variable and unpredictable future climate.

## CONFLICT OF INTEREST

None declared.

## AUTHOR CONTRIBUTIONS

NNM and SB designed the experiment. ZKN and TNK provided the populations. NNM performed the experiment. NNM and SB analyzed the data and wrote the first draft of the manuscript, which was improved and revised according to the comments of all authors.

## Data Availability

Data is available from the Dryad Digital Repository: https://doi.org/10.5061/dryad.cm042k3.
